# Antioxidative and antidiabetic effects of germinated rough rice extract in 3T3-L1 adipocytes and C57BLKS/J-*db/db* mice

**DOI:** 10.29219/fnr.v63.3603

**Published:** 2019-11-28

**Authors:** Youn Ri Lee, Sang Hoon Lee, Gwi Yeong Jang, Yoon Jeong Lee, Min Young Kim, Yun-Bae Kim, Junsoo Lee, Heon Sang Jeong

**Affiliations:** 1 Department of Food and Nutrition, Daejeon Health Sciences College, Daejeon, Korea; 2 Department of Agrofood Resources, National Academy of Agricultural Science, Rural Development Administration, Wanju, Korea; 3 Department of Herbal Crop Research, National Institute of Horticultural and Herbal Science, Rural Development Administration, Eumseong, Korea; 4 Department of Food Science and Biotechnology, Chungbuk National University, Cheongju, Korea; 5 College of Veterinary Medicine, Chungbuk National University, Cheongju, Korea

**Keywords:** germinated rough rice, antioxidant property, type II diabetes, glucose uptake, C57BLKS/J-db/db mice

## Abstract

**Background:**

To overcome disadvantages of germinated brown rice, we germinated rough rice and tested effects of its useful ingredients on prevention of diabetes.

**Objective:**

This study investigated the *in vitro* antioxidant and *in vivo* antidiabetic effects of rough rice (*Oryza sativa* L.) with hulls, before and after germination. Rough rice was germinated for 4 days and extracted with water at 120°C.

**Design:**

This study measured antioxidants and antioxidative effects and inhibitory activities against α-amylase and α-glucosidase of rough rice before and after germination and investigated antidiabetic effects of rough rice through animal experiments.

**Results:**

All these factors increased after germination. Also, α-amylase and α-glucosidase inhibition and glucose uptake by 3T3-L1 adipocytes were significantly increased after germination. Oral administration of the germinated rough rice extract for 8 weeks significantly increased insulin levels and decreased blood glucose levels in a C57BLKS/J-*db/db* mice model. Immunohistochemical analysis showed that germinated rough rice effectively protected against liver, kidney, and pancreatic tissue damage.

**Discussion:**

Useful ingredients in germinated rough rice could be used to prevent diabetes.

**Conclusions:**

These results suggest that germinated rough rice extract had a beneficial effect on diabetes by increasing the antioxidant activity and further purification studies are necessary to elucidate the mechanism of the extract’s antidiabetic activity.

## Popular scientific summary

- Effect of germinated rough rice extracted with hot water on prevention of type 2 diabetes was examined.
- Useful ingredients extracted from germinated rough rice could lower glucose level.
- Marker substances and mechanism of useful ingredients in germinated rough rice need further research to develop various foods using these ingredients.

Diabetes mellitus is a metabolic disease characterized by hyperglycemia resulting from defects in insulin secretion and/or insulin action, and 90–95% of all diabetic cases are of type 2 (1). Type 2 diabetes (noninsulin-dependent diabetes mellitus, NIDDM) is mostly characterized by pancreatic β-cell dysfunction and decreased insulin sensitivity. Not only do over 80% of NIDDM patients have insulin resistance, but NIDDM also is characterized by the continuous and accelerating loss of β-cell mass and function (2).

Germination is an effective and common process used to improve the nutritional quality of cereals (3). The germination process is affected by external factors, such as germination time and the absence or presence of light, both of which can aid or inhibit germination in relation to the reserve (nutrition content) within the seed (4). Activated hydrolytic enzymes in germinated cereal grains decompose starch, nonstarch polysaccharides, and proteins, which leads to an increase in oligosaccharides and amino acids in cereals (5). Decomposition of high molecular weight polymers during germination also leads to the generation of biofunctional substances, as well as improved organoleptic qualities due to the softened texture and increased flavor (6). Furthermore, germination increases the content of essential amino acids, such as lysine, methionine, γ-oryzanol, and γ-aminobutyric acid (7, 8). Changes in the phenolic content and radical scavenging activity have also been reported (9). It is important to reduce lipid peroxides, oxidative stress, and blood glucose when trying to control diabetes to reduce the onset of diabetic complications. Thus, it is necessary to continuously search for plant materials that decrease high blood glucose levels and oxidative stress. A natural approach to treating type 2 diabetes may also help reduce adverse reactions to therapeutic agents, such as oral hypoglycemic drugs.

This study was conducted to evaluate the *in vitro* antioxidant properties and to verify the increase in glucose absorption by enhancing insulin sensitivity in 3T3-L1 cells. In addition, the antidiabetic efficacy of germinated rough rice extract was investigated in C57BLKS/J-*db/db* mice, a model of type 2 diabetes.

## Materials and methods

### Germination and extract preparation

The rough rice (*Oryza sativa* L. Cv. Ilpum,) with hull was provided by the National Institute of Crop Science, Rural Development Administration, Korea. The rough rice was soaked in tap water for 3 days at room temperature and the soaking water was changed every 24 h. The soaked rough rice was germinated at 37°C and relative humidity of 80% until the length of the sprouts was 15–25 mm. After germination, the rough rice was dried at 55°C for 24 h and then milled (Micro hammer cutter mill type-3; Culatti AG, Zurich, Switzerland) to a particle size <180 μm. The germinated rough rice was extracted with distilled water at 120°C for 3 h and wrapped in a polyethylene film pouch to have their dried weight as 1%. During the 8-week experimental period, the substance was stored in the refrigerator and the package was opened right before use. The ungerminated rough rice did not go through the germination process.

### Total polyphenol and flavonoid contents

The total polyphenol content of the extracts was determined using the Folin-Ciocalteu assay (10). In a 1.5 mL microtube, 1 mL of 2% Na_2_CO_3_ solution was mixed with 50 μL of the sample. After 3 min, 50 μL of 2 N Folin-Ciocalteu’s phenol reagent (Sigma-Aldrich, St. Louis, MO, USA) was added and mixed. After exactly 30 min, the absorbance was determined at 750 nm with a UV–Vis spectrophotometer (UV-1650PC; Shimadzu, Kyoto, Japan). The total polyphenol contents of the extracts were expressed as mg of gallic acid equivalents (GAE) per g of dry weight.

The total flavonoid contents were measured according to a modification of the method of Dewanto et al. (10). An aliquot (125 μL) of the extracts or standard solution of catechin was added to 1.5 mL microtube containing 500 μL of distilled water and 37.5 μL of 5% NaNO_2_ was added. After 5 min, 75 μL of 10% A1C1_3_ was added. After 6 min, 250 μL of 1 M NaOH was added and mixed. After exactly 11 min, the absorbance was determined at 510 nm with a UV–Vis spectrophotometer. The total flavonoid content was expressed as mg catechin equivalents (CE) per g of dry weight.

### Phenolic acid composition

The determination of the major phenolic acids in the rough rice extracts was carried out on a reversed-phase high-performance liquid chromatography system (Acme 9000 system, Younglin Co., Anyang, Korea) coupled with a UV–Vis detector, binary pump, and an autosampler (11). The HPLC column was a Mightysil RP-18 (250 × 4.6 mm, 5 μm; Kanto Co., Ltd., Kyoto, Japan). The mobile phases consisted of A (0.1% acetic acid in acetonitrile) and B (0.1% acetic acid in water). A gradient established in our laboratory was applied as follows: 0–2 min, 8–10% A; 2–27 min, 10–30% A; 27–50 min, 30–90% A; 50–51 min, 90–100% A; 51–60 min, 100% A; 60–61 min, 100–8% A; and 61–70 min, 100–8% A. The injection volume of the sample was 20 μL and the flow rate was 1.0 mL/min. The monitor wavelength was set at 280 nm. Before analysis, all samples were filtered through a 0.25 μm membrane filter (Millipore, Billerica, MA, USA). The samples were quantified by comparing the retention times with known authentic standards.

### Antioxidant activities

The antioxidant activity was assessed quantitatively using the 2,2-diphenyl-1-picrylhydrazyl (DPPH) method (9). A 0.1 mM DPPH solution was prepared in ethanol and further diluted in ethanol to obtain an absorbance of 1.4–1.5 at 520 nm. About 0.2 mL of extract solution or standard solution of l-ascorbic acid was added to the ethanolic solution of DPPH. The mixture was shaken vigorously and kept at room temperature for 30 min in the dark. The absorbance was determined at 520 nm using a UV–Vis spectrophotometer (UV-1650PC; Shimadzu, Kyoto, Japan). The DPPH radical scavenging activity was expressed as mg of l-ascorbic acid equivalents per g of dry weight.

The 2,2′-azinobis-[3-ethylbenzothiazoline-6-sulfonic acid] (ABTS) radical scavenging activity assay was carried out using a modified method of Lee et al. (9). Briefly, ABTS was dissolved in distilled water to 7 mM. The ABTS stock solution was then obtained by mixing the 7 mM ABTS solution with 2.45 mM potassium persulfate solution and leaving it in the dark at room temperature for 12 h before use. The ABTS radical solution was diluted with distilled water and adjusted to an absorbance of 1.400 ± 0.020 at 735 nm. The assay was performed using 96-well microplates. The extract (0.05 mL) was added to 1 mL of the ABTS radical solution for use in each reaction. The absorbance was measured at 735 nm. The ABTS antioxidant activity was expressed as mg of l-ascorbic acid equivalents per g of dry weight.

The reducing power of the extracts was determined according to the method of Aktumsek et al. (12). Equal volumes (250 μL) of the extracts, 0.2 M sodium phosphate buffer (pH 6.6), and 1% potassium ferricyanide [K_3_Fe (CN)_6_] were mixed. The mixture was shaken vigorously and kept at 50°C for 20 min in the dark. Then, the mixture was added to 1% trichloroacetic acid (CCl_3_COOH, w/v). The absorbance at 700 nm was determined using a UV–Vis spectrophotometer (UV-1650PC; Shimadzu, Kyoto, Japan). The reducing power was expressed as the absorbance at 700 nm with concentration of 5 mg/mL.

The chelating effect was determined according to the method of Aktumsek et al. (12). Briefly, 1 mL (10 mg/mL) of the extracts in distilled water was added to 0.1 mL of distilled water and a solution of 2 mM FeCl_2_ (0.05 mL). The reaction was initiated by the addition of 5 mM ferrozine (0.2 mL). Then, the mixture was shaken vigorously and left at room temperature for 10 min. The absorbance of the solution was measured spectrophotometrically at 562 nm. The percent inhibition of the ferrozine–Fe^2+^ complex formation was calculated.

### a-Amylase and a-glucosidase inhibitory activities

The α-amylase inhibitory activity was measured according to Lim et al. (13) with slight modification. Briefly, sample extracts (125 μL) and 62.5 μL of 200 mM potassium phosphate buffer (pH 6.8) containing 62.5 μL of porcine pancreatic α-amylase (12 unit/mL) were preincubated at 37°C for 10 min. Then, 125 μL of 1% starch solution in 200 mM sodium phosphate buffer (pH 7.0) was added to each tube. The reaction mixtures were incubated at 37°C for 5 min and stopped with 125 μL of DNS (3,5-dinitrosalicylic acid and 30% sodium potassium tartrate in 0.5 M NaOH) color reagent. Thereafter, the mixture was incubated in a boiling water bath for 15 min and cooled to room temperature. The reaction mixture was diluted by adding distilled water and the absorbance was measured at 540 nm.

The α-glucosidase inhibitory activity was measured according to the modified method of Gao and Kawabata (14). Crude enzyme solution prepared from rat intestinal acetone powder (Sigma-Aldrich) was used as a source of small intestinal α-glucosidases. Rat intestinal acetone powder was hand-homogenized using ice-cold 0.9% NaCl solution. A 50 μL aliquot of sample was preincubated with crude enzyme solution (100 μL) as a source of α-glucosidase at 37°C for 10 min. The substrate solution (*p*-NPG: 5 mM, 50 μL) in 0.1 M sodium phosphate buffer (pH 6.8) was then added to the reaction mixture and incubated at 37°C for 20 min. The absorbance of liberated *p*-nitrophenol was measured at 405 nm using a spectrophotometer (UV-1650PC, Shimadzu, Kyoto, Japan).

### Cell culture and adipocytes differentiation

The 3T3-L1 cells were cultured and differentiated according to the method of Lee et al. (15). The prefibroblasts were cultured in high-glucose Dulbecco’s modified Eagle’s medium (DMEM) with 10% bovine serum (BS) and 1% antibiotic–antimycotic at 37°C in 5% CO_2_. The cells were seeded at 4.0 × 10^4^/well in 6-well plates. They were induced to differentiation using high-glucose DMEM with 10% fetal bovine serum (FBS), 1% antibiotic, 5 ng/mL of insulin, 1 mM 3-isobutyl-1-methylxanthine (IBMX), and 1 mM dexamethasone in 2 days after reaching confluence and then they were changed to high-glucose DMEM with 10% FBS and 1% antibiotic 2 days after the initiation of differentiation. The differentiated fibroblasts were used for 2-deoxyglucose-uptake measurement.

### Glucose-uptake assay

2-Deoxyglucose uptake was evaluated according to the modified method of Park et al. (16) using a 2-deoxyglucose uptake measurement kit (Cosmo Bio, Tokyo, Japan). The media of the differentiated fibroblasts were changed to high-glucose DMEM with 1% antibiotic and the differentiated fibroblasts were incubated for 6 h. The differentiated fibroblasts were washed with Krebs-Ringer phosphate-HEPES (KRPH) buffer (pH 7.5) and 2% BS albumin (in KRPH buffer) was added to each sample and the cells were incubated for 20 min. Then, they were washed with phosphate buffer saline (PBS) with 200 μM phloretin, 10 mM Tris–HCl buffer solution (3 mL) was added and the cells were incubated at 80°C for 15 min, followed by centrifugation at 15,000 × *g* for 20 min. The supernatants were diluted 5-fold with sample diluent buffer and evaluated using 2-deoxyglucose uptake kit.

### Animals

Five-week-old C57BLKS/J-*db/db* mice were purchased from Hyochang Science (Daegu, Korea). The animals were individually housed in stainless steel cages in a room maintained at 20–22°C and 50±10% relative humidity with a 12-h light/dark cycle. All the mice were fed a pelletized commercial chow diet and sterile water *ad libitum* for 7 days. The use of laboratory animals was approved by the Committees of Animal Experiments, Chungbuk National University and managed according to the Guidelines on Maintaining and Use of Laboratory Animals (PE0701-1). The body weights and rough rice extract were measured daily and weekly, respectively. The experimental groups were normal control (NC), diabetic control (DC), diabetic mellitus before germinated rough rice extract (BG), and diabetic mellitus after germinated rough rice extract (AG). The NC and DC groups drank water, while the experimental diabetic groups consumed rough rice extract instead of water, as needed, during the 8 weeks. At the end of the experimental period of 8 weeks, the mice were anesthetized with an i.p. injection of 1% ketamine hydrochloride following a 12 h fast and subsequent weighing. Blood was collected from the abdominal aorta of the euthanized animals into a heparinized syringe and plasma was obtained by centrifugation at 3,000 rpm at 4°C for 20 min. The liver, kidney, and pancreas were removed and perfused with cold physiological saline. The excised organs were blotted dry and weighed. All samples prepared were stored at –70°C until analyzed.

### Measurement of glucose tolerance test

Seven days before the postmortem examination, a glucose tolerance test was conducted. The animals were fasted for 16 h and the blood sugar levels were measured right before the oral administration of 1.5 g/kg of glucose and then 15, 30, 60, 90, and 120 min after the administration and the changes were recorded. The area under the curve (AUC) of the graph was calculated for comparison.

### Measurement of fasting blood glucose and serum insulin levels

Changes in the fasting blood glucose level were measured while supplying the test substance once every 2 weeks for 8 weeks. The values were measured at baseline (0 weeks), 4, and 8 weeks. Blood was collected from the tip of the tail vein and the fasting blood glucose level was measured using a glucometer (Glucotrend, Mannheim, Germany). Serum insulin was analyzed with an enzyme-linked immunosorbent assay (ELISA) kit (AKRIN-011H; Shibayagi Co., Gunma, Japan) following the manufacturer’s protocol. The insulin concentration was measured using a luminescent microplate reader at 450 nm.

### Histopathological examination

The liver, kidney, and pancreas were fixed in formalin, underwent general tissue pathology processing, and were made into slides by embedding in paraffin. The histologic slides were stained with hematoxylin–eosin and examined for the presence and degree of pathological abnormalities using an optical microscope.

### Statistical analyses

The results were expressed as the mean ± SE of the 10 animals. Statistical comparison of the differences between the groups was carried out using one-way ANOVA tests, followed by Duncan’s multiple range test, using the SPSS statistical software package (Version 12.0, SPSS Inc., Chicago, IL, USA).

## Results and discussion

### Total polyphenol and flavonoid content

The total polyphenol and flavonoid content of the rough rice extract before and after germination are shown in Table 1. The total polyphenol and flavonoid content increased from 4.11 mg GAE/g and 2.14 mg CE/g before germination to 9.82 mg GAE/g and 3.85 mg CE/g after germination, respectively (*P* < 0.001). Lee et al. (9) reported that the total phenolic content of several rice cultivars increased from 2.1–4.9 mg/g before germination to 3.1–7.9 mg/g after germination. Also, the phenolic contents of rough rice increased as the germination periods increased (17–19). The higher total phenolic content of the germinated rice extracts compared to that in nongerminated rice extracts could be due to the biosynthesis of phenolic compounds caused by enzyme hydrolysis during germination (20). Phenolic compounds are the most important contributors to the antioxidant capacity of cereal grains and play an essential role in the prevention and control of degenerative diseases (21).

**Table 1:** Changes in total polyphenol and flavonoid content of rough rice extract before and after germination

Phenolic compounds	Before germination	After germination
Total polyphenol content (mg gallic acid eq/g)	4.11 ± 0.03	9.82 ± 0.08***
Total flavonoid content (mg catechin eq/g)	2.14 ± 0.01	3.85 ± 0.03***

The results are expressed as the mean ± SD of triplicate samples.

*** *P* < 0.001; significantly different before and after germination by Student’s *t*-test.

### Phenolic acid composition

Changes in the phenolic acid composition of the rough rice extract before and after germination are shown in Table 2. Twelve kinds of phenolic acids were detected and the major phenolic acids were *trans*-cinnamic acid (11.09 mg/100 g), salicylic acid (3.91 mg/100 g), *p*-coumaric acid (3.60 mg/100 g), naringenin (3.51 mg/100 g), and kaempferol (3.34 mg/100 g). With the exception of naringin, the phenolic acid contents of rough rice extract significantly increased after germination (*P* < 0.001). Specifically, the ferulic acid (4.9-fold), myricetin (4.9-fold), caffeic acid (3.9-fold), and gallic acid (3.9-fold) contents were greatly increased after germination. Kim et al. (19) reported that the major phenolic acid in rough rice was *trans*-cinnamic acid and most phenolic acid content increased after germination. Phenolic acids are widely distributed throughout the plant kingdom and have a wide range of structures and molecular weights. Also, phenolic acids are known to have physiological activities, such as antioxidant, anticancer, antidiabetic, and antibacterial effects through a combination of hydroxyl group with protein and macromolecules (22). Many types of phenolic acids are found in conjunction with cell wall polysaccharides, starch, and protein. However, phenolic acid increases after germination due to the activation of α-amylase and protease (23).

**Table 2:** Changes in phenolic acid composition of rough rice extract before and after germination

Phenolic acids	Content (mg/100 g)	
	Before germination	After germination
Caffeic acid	0.54 ± 0.01	2.12 ± 0.10***
Catechin	2.83 ± 0.13	8.18 ± 0.35***
Chlorogenic acid	0.11 ± 0.00	0.34 ± 0.01***
*trans*-Cinnamic acid	11.09 ± 0.40	37.33 ± 1.40***
*p*-Coumaric acid	3.60 ± 0.14	11.15 ± 0.34***
Ferulic acid	2.38 ± 0.04	11.72 ± 0.32***
Gallic acid	0.81 ± 0.03	3.21 ± 0.11***
Kaempferol	3.34 ± 0.35	8.86 ± 0.40***
Myricetin	0.64 ± 0.01	3.15 ± 0.11***
Naringenin	3.51 ± 0.11	12.04 ± 0.26***
Naringin	2.22 ± 0.10	2.10 ± 0.06^NS^
Salicylic acid	3.91 ± 0.13	11.77 ± 0.45***

The results are expressed as the mean ± SD of triplicate samples.

*** *P* < 0.001; significantly different before and after germination by Student’s *t*-test.

### Antioxidant activities

The changes in antioxidant activities of the rough rice extract before and after germination are shown in Table 3. The DPPH and ABTS radical scavenging activities of rough rice extract significantly (*P* < 0.001) increased from 3.75 mg TE/g and 18.58 mg AE/g before germination to 6.54 mg TE/g and 28.73 mg AE/g after germination, respectively. Also, the reducing power and iron chelating effect of the rough rice extract increased to 0.745 and 85.46% by germination, respectively (*P* < 0.001). Lee et al. (9) reported that the reducing power, DPPH radical, superoxide radical, and hydroxyl radical scavenging activities of several rice cultivars increased following germination. In a similar study, the antioxidant activities of rough rice extracts increased to 4 day of germination period (19). In general, the phenolic contents were positively correlated with antioxidant activity due to their hydrogen-donating abilities. The strong positive linear correlations between free radical scavenging activities and polyphenolic compound concentrations in various food materials are well known (17–19). These reports suggest that the radical scavenging capacities of extracts are mostly affected by the presence and position of phenolic hydroxyl groups. The radical scavenging activity of the phenolic compounds depends on their molecular structure, that is, on the availability of phenolic hydrogen and the stabilization of the resulting phenoxyl radicals that are formed by hydrogen donation.

**Table 3:** Changes in antioxidant activities of rough rice extract before and after germination

Antioxidant activity	Before germination	After germination
DPPH radical scavenging activity (mg Trolox eq/g)	3.75 ± 0.06	6.54 ± 0.07***
ABTS radical scavenging activity (mg ascorbic acid eq/g)	18.58 ± 0.21	28.73 ± 0.35***
Reducing power (*A*_700_ at 5 mg/mL)	0.447 ± 0.014	0.745 ± 0.021***
Iron chelating effect (% at 10 mg/mL)	56.27 ± 0.94	85.46 ± 1.24***

The results are expressed as the mean ± SD of triplicate samples.

*** *P* < 0.001; significantly different before and after germination by Student’s *t*-test.

### α-Amylase and α-glucosidase inhibitory activities

The changes in α-amylase and α-glucosidase inhibitory activities of the rough rice extract before and after germination are shown in Table 4. The α-amylase inhibitory activity increased from 34.37 to 52.16% at a concentration of 1 mg/mL after germination and α-glucosidase inhibition increased from 10.14 to 66.04% at 10 mg/mL following germination (*P* < 0.001). Kim et al. (24, 25) reported that α-amylase and α-glucosidase inhibitory activities increased after germination and increased to 4 days of germination period. Consistent with our results, higher α-amylase and α-glucosidase inhibitory activities were reported in germinated barley, sorghum, oat, rye, brown rice, wheat, and buckwheat grains (26, 27). Many researchers suggest that phenolic compounds present in grains may play a key role in the inhibition of α-amylase and α-glucosidase (24–27). Shobana et al. (28) found that finger millet seed coat polyphenols inhibited α-amylase and α-glucosidase in a dose-dependent manner and mass spectra of the finger millet extract showed the presence of naringenin, kaempferol, luteolin glycoside, and (+)-catechin/(–)-epicatechin. In addition, naringenin and kaempferol were the major phenolic acids detected in raw and germinated rough rice extracts in the present study (Table 2), indicating their potential role in the inhibition of α-amylase and α-glucosidase. Inhibition of these enzymes by phenolics suggests that these germinated rough rice may help reduce the release and absorption of glucose in the small intestine, and thus provide a beneficial effect on diabetes by ameliorating postprandial glycemic responses.

**Table 4:** Changes in α-amylase and α-glucosidase inhibitory activity of rough rice extract before and after germination

Enzyme inhibition	Before germination	After germination
α-Amylase (% at 1 mg/mL)	34.37 ± 1.25	52.16 ± 1.07***
α-Glucosidase (% at 10 mg/mL)	10.14 ± 0.11	66.04 ± 1.75***

The results are expressed as the mean ± SD of triplicate samples.

*** *P* < 0.001; significantly different before and after germination by Student’s *t*-test.

### Antidiabetic effect on 3T3-L1 adipocytes

**Fig. 1:**
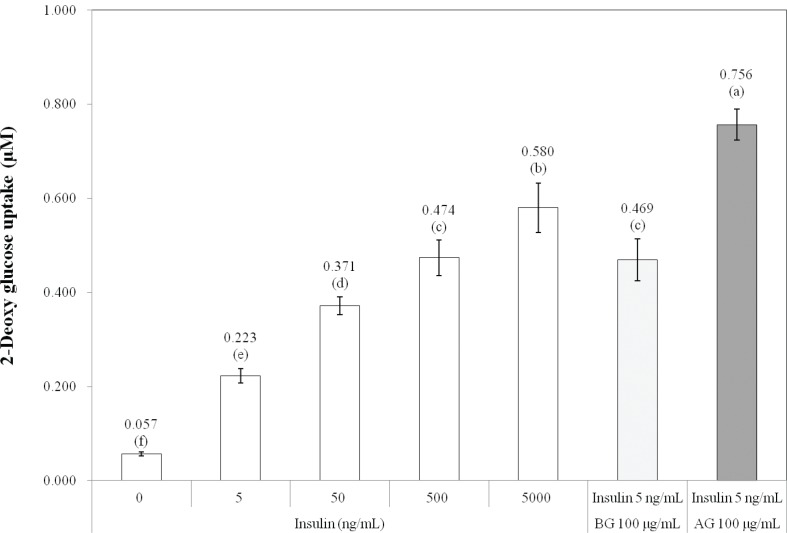
Effects of insulin concentrations and rough rice extract before (BG) and after germination (AG) on glucose uptake by 3T3- L1 adipocytes. ^a–f^Significant differences by Duncan's multiple range test (*P* < 0.05).

**Fig. 2:**
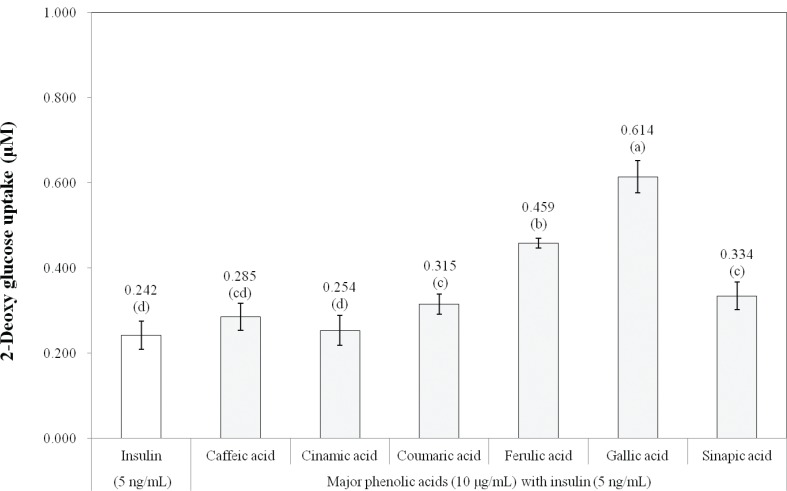
Effects of major phenolic acids in germinated rough rice extract on glucose uptake by 3T3-L1 adipocytes. ^a–d^Significant differences by Duncan’s multiple range test (*P* < 0.05).

The insulin-mediated glucose uptake increased in a dose-dependent manner up to 5,000 ng/mL insulin in 3T3-L1 adipocytes (Fig. 1). Insulin sensitizers enhance insulin action only when insulin exists. In the present study, a minimum amount of insulin (5 ng/mL) was used to evaluate the *in vitro* antidiabetic effect of the rough rice extracts and major phenolic acids. In addition to 5 ng/mL insulin, treatment of rough rice extract before and after germination showed increased insulin-stimulated glucose uptake in 3T3-L1 adipocytes, compared to treatment with 5 ng/mL insulin (*P* < 0.05). Specifically, following treatment with 100 μg/mL of rough rice extract after germination and 5 ng/mL insulin, the glucose uptake increased as much as that seen following treatment with 5,000 ng/mL insulin (*P* < 0.05). Treatment with 10 μg/mL of gallic acid, ferulic acid, sinapic acid, and *p*-coumaric acid and 5 ng/mL insulin increased insulin-stimulated glucose uptake in 3T3-L1 adipocytes, compared to treatment with 5 ng/mL insulin alone (Fig. 2, *P* < 0.05). In particular, the treatment of gallic acid and ferulic acid increased the insulin-stimulated glucose uptake as much as that increased by 500 and 5,000 ng/mL insulin, respectively (*P* < 0.05). Jung et al. (29) reported that ferulic acid, *p*-coumaric acid, and sinapic acid from rice bran extract stimulated glucose uptake and glucokinase activity in HepG2 cells. Cinnamic acid and gallic acid were reported to increase glucose uptake and regulate glucose transport in 3T3-L1 cells via the activation of GLUT4 and PI3-kinase (30, 31).

### Antidiabetic effect in C57BLKS/J-db/db mice

No signs of toxicity or death were recorded during the daily administration of rough rice extract for 8 weeks. The changes in body weight, feed intake, water/rough rice extract consumption, and relative organ weight are shown in Table 5. A significant (*P* < 0.05) difference in weight was observed between the DC and NC groups; however, there are no significant differences between the type 2 diabetic *db/db* mice groups after 8 weeks. The DC group consumed 1.7-fold more food than that the NC group, but no significant difference in food intake was observed between the BG and AG groups. The DC group drank 5-fold more than the NC group, but no difference was observed between the BG and AG groups. Diabetic mice generally drank 5–8-fold more water than did the normal mice. The weights of the livers and kidneys in the group fed rough rice extract were decreased compared to those in the DC group and a significant difference was observed in the AC group. The weights of the liver and kidney tissues in the DC group were higher than those in the NC group, but tended to decrease following the intake of the AG group. Polydipsia, polyuria, and polyphagia are typical symptoms observed in diabetes (32). Yamanaka et al. (33) reported that treatment with rosiglitazone increased the body weight of *db/db* mice. Rosiglitazone, an antidiabetic drug in the thiazolinedion class, induces adipogenesis and causes body-wide lipid repartitioning by increasing the adipose triglyceride (TG) content, thereby lowering circulating free fatty acids (FFA), TG, and glucose levels, which are associated with increased insulin sensitivity in the liver, muscles, and other organs.

**Table 5:** Effects of rough rice extract for 8 weeks on changes in body weight, food intake, water/extract intake, and relative organ weights in type II diabetic C57BLKS/J-*db/db* mice

Group	Body weight (g)		Food intake (g/day)	Water/extract intake (mL/day)	Liver (%/BW)	Kidney (%/BW)
	Initial	Final				
NC	28.45±1.58^1^^b^	31.12±1.37^2^^b^	4.27±0.288^d^	6.54±0.26^b^	4.26±0.45^b^	0.59±0.01^a^
DC	42.12±2.31^a^	50.42±5.45^a^	7.38±0.26^a^	30.73±2.25^a^	5.97±0.19^a^	0.53±0.05^a^
BG	42.08±2.12^a^	53.12±5.12^a^	6.46±0.95^bc^	29.74±8.55^a^	5.64±0.32^bc^	0.44±0.03^b^
AG	43.37±2.04^a^	58.51±6.12^a^	5.78±0.52^c^	24.29±3.60^a^	5.31±0.17^c^	0.39±0.01^c^

1 Each value is expressed as mean±SE.

2 Means with different superscript letters in the same column are significantly different at *P* < 0.05 by one-way ANOVA.

Different letters (a-c) above the bars indicate significant different (*P* < 0.05) among the different group..

The blood glucose and insulin levels are shown in Table 6. The blood glucose level in the NC group was 109.12 mg/dL, while that in the DC group prior to extract administration was 384.12 mg/dL. The blood glucose of the DC group injected for 8 weeks maintained the highest point. The hypoglycemic effect was seen after 4 weeks in the AG group, rather than BG. Table 7 shows the glucose tolerance test results. The glucose level increased to 240 mg/dL 30 min after glucose administration, decreased, then returned to normal within 120 min in normal animals. However, the glucose level increased drastically to 795 mg/dL 30 min after glucose administration to diabetic rats, decreased gradually thereafter, and remained at 680 mg/dL for 120 min. The maximum blood sugar concentration was lower and the reduction more marked in animals fed in the AG and BG groups, compared to the levels in diabetic animals. The area under the glucose curve indicated that the greatest effects occurred in the AG group. The AUC (0–120 min) for blood glucose levels in the NC (23,860) group was the lowest over a 120-min period of intervention, while the DC (88,263) group had the highest and the BG (77,318) and AG (72,048) groups produced lower AUCs than the DC group. By the end of the experiment, the AUC of the BG group was higher than that of the AG group, suggesting that the high glycemic index in the BG group maintained elevated blood glucose levels more than the AG or the NC groups.

**Table 6:** Effect of germinated rough rice extract supplementation on blood glucose and serum insulin in C57BLKS/J-*db/db* mice

Group	Blood glucose (mg/dL)			Serum insulin (ng/mL)
	0 week	4 week	8 week	
NC	109.12±6.03^1^^a^	104.62±7.55^2^^a^	88.00±4.13^a^	0.23±0.22^a^
DC	384.12±46.59^b^	432.87±47.10^b^	507.25±22.49^c^	1.22±0.32^b^
BG	381.25±39.63^b^	594.00±26.74^c^	452.87±40.16^bc^	1.58±0.25^b^
AG	386.33±40.30^b^	400.07±47.56^b^	396.00±41.49^b^	2.57±0.25^c^

1 Each value is expressed as mean±SE.

2 Means with different superscript letters in the same column are significantly different at *P* < 0.05 by one-way ANOVA.

Different letters (a-c) above the bars indicate significant different (*P* < 0.05) among the different group.

**Table 7:** Effect of germinated rough rice extract on glucose tolerance tests in type 2 diabetic C57BLKS/J-*db/db* mice

Group	Fasting	30 min	60 min	90 min	120 min
NC	86.75±2.78^1^^a^	242.3±23.3^2^^a^	197.7±19.8^a^	177.0±14.1^a^	143.3±17.8^a^
DC	555.6±13.2^b^	795.2±21.7^b^	760.5±42.3^c^	671.8±37.5^b^	680.3±41.2^c^
BG	489.3±53.4^b^	743.8±32.2^b^	585.1±40.5^b^	577.3±56.7^b^	526.3±58.8^b^
AG	471.7±31.6^b^	712.0±41.7^b^	616.0±25.2^b^	573.7±52.3^b^	520.0±28.8^b^

1 Each value is expressed as mean±SE.

2 Means with different superscript letters in the same column are significantly different at *P* < 0.05 by one-way ANOVA.

Different letters (a-c) above the bars indicate significant different (*P* < 0.05) among the different group.

Declining β-cell function has been identified as a major factor associated with progressively higher plasma glucose levels (2). However, most of the antidiabetic drugs did not prevent pancreatic atrophy and function in patients or the NIDDM animal model (34). The germinated rough rice extracts prevented atrophy of the pancreatic islets of Langerhans cells in type 2 diabetic mice and promoted insulin secretion by protecting β-cells, even at a concentration of 1%. This increase in insulin levels decreased blood sugar concentrations in C57BLKS/J-*db/db* mice, which do not have a good tolerance for insulin, thereby significantly suppressing feed intake, one of the major symptoms of diabetes. The effects of the rice extract on lipid metabolism mitigated the cytopathic effects and histological damage in the liver and kidneys. Therefore, this rice could be developed as a functional health food to induce hypoglycemia in diet therapy for patients with diabetes. In conclusion, these results suggest that germinated rough rice extract had a beneficial effect on diabetes. The antidiabetic activity may be attributed to polyphenols and further separation and purification studies are necessary to ascertain the mechanism involved in the antidiabetic effects of germinated rough rice extract.

### Histopathological evaluation of C57BLKSJ-db/db mice

**Fig. 3:**
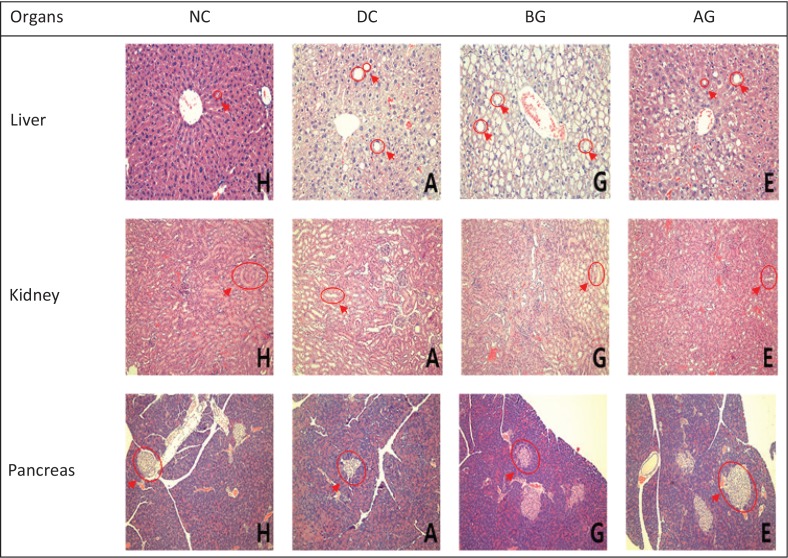
Histological findings based on hematoxylin–eosin staining of liver, kidney, and pancreas in C57BLKS/J-*db/db* mice. Liver, kidney, and pancreas tissues were formalin-fixed and paraffin-embedded. Four-micron-thick tissue sections were prepared and stained with H&E (**×**200). H, NC (normal control); A, DC (diabetic control); G, before germinated rough rice extract (BG); E, after germinated rough rice extract (AG).

The livers of the C57BLKS/J-*db/db* mice showed serous fatty and vacuolar degeneration (Fig. 3). The liver surface in the positive control group (positive group) deteriorated due to fat and lipid deposition. We detected necrosis and infiltration of inflammatory cells. However, hepatocyte accumulation was inhibited in the AG group. These cytopathic effects were highly moderated in the AG group; therefore, the rice inhibited hepatic fat accumulation through lipid metabolism. Kidney tissue also revealed expanded renal tubules and local degeneration in obese diabetic mice. Such lesions also appeared to be mitigated in the AG group. The β-cells in the islets of Langerhans were significantly decreased in size in the DC group, compared to those of the NC group. The AG group had a hypoglycemic effect for 8 weeks, suggesting that the protective effect in the β-cells was related to the promotion of insulin secretion. Recently, the destruction of pancreatic islets was reported to be one of the main sources of problems in NIDDM patients (35).

## Conflict of interest

No potential conflict of interest was reported by the authors.

## Funding

This research was supported by the High Value-Added Food Technology Development Program (112077-03-SB010), Ministry of Agriculture, Food and Rural Affairs, Korea.
